# Deep-Learning Segmentation of Epicardial Adipose Tissue Using Four-Chamber Cardiac Magnetic Resonance Imaging

**DOI:** 10.3390/diagnostics12010126

**Published:** 2022-01-06

**Authors:** Pierre Daudé, Patricia Ancel, Sylviane Confort Gouny, Alexis Jacquier, Frank Kober, Anne Dutour, Monique Bernard, Bénédicte Gaborit, Stanislas Rapacchi

**Affiliations:** 1 Aix-Marseille Univ, CNRS, CRMBM, 13005 Marseille, France; s.confort-gouny@univ-amu.fr (S.C.G.); alexis.jacquier@ap-hm.fr (A.J.); frank.kober@univ-amu.fr (F.K.); monique.bernard@univ-amu.fr (M.B.); 2 APHM, Hôpital Universitaire Timone, CEMEREM, 13385 Marseille, France; 3 Department of Radiology, APHM, La Timone Hospital, 13005 Marseille, France; patricia.ancel@univ-amu.fr; 4 Aix-Marseille Univ, INSERM, INRAE, C2VN, 13005 Marseille, France; anneevesophie.meyer@ap-hm.fr (A.D.); benedicte.gaborit@ap-hm.fr (B.G.); 5 Department of Endocrinology, Metabolic Diseases and Nutrition, Pôle ENDO, APHM, Hôpital Nord, Chemin Des Bourrely, 13005 Marseille, France

**Keywords:** epicardial adipose tissue quantification, automatic segmentation, cine four-chamber, fully convolutional networks, machine learning

## Abstract

In magnetic resonance imaging (MRI), epicardial adipose tissue (EAT) overload remains often overlooked due to tedious manual contouring in images. Automated four-chamber EAT area quantification was proposed, leveraging deep-learning segmentation using multi-frame fully convolutional networks (FCN). The investigation involved 100 subjects—comprising healthy, obese, and diabetic patients—who underwent 3T cardiac cine MRI, optimized U-Net and FCN (noted FCNB) were trained on three consecutive cine frames for segmentation of central frame using dice loss. Networks were trained using 4-fold cross-validation (*n* = 80) and evaluated on an independent dataset (*n* = 20). Segmentation performances were compared to inter-intra observer bias with dice (DSC) and relative surface error (RSE). Both systole and diastole four-chamber area were correlated with total EAT volume (r = 0.77 and 0.74 respectively). Networks’ performances were equivalent to inter-observers’ bias (EAT: DSC_Inter_ = 0.76, DSC_U-Net_ = 0.77, DSC_FCNB_ = 0.76). U-net outperformed (*p* < 0.0001) FCNB on all metrics. Eventually, proposed multi-frame U-Net provided automated EAT area quantification with a 14.2% precision for the clinically relevant upper three quarters of EAT area range, scaling patients’ risk of EAT overload with 70% accuracy. Exploiting multi-frame U-Net in standard cine provided automated EAT quantification over a wide range of EAT quantities. The method is made available to the community through a FSLeyes plugin.

## 1. Introduction

Epicardial adipose tissue (EAT) is a visceral fat depot surrounding the heart between the myocardium and the pericardium [1]. Its volume quantification holds potential as a novel biomarker for risks of coronary heart disease [2]. Pericardial fat, merging EAT and paracardial (PAT) fat, has been studied in the past in association with atherosclerotic disease [3] but these results have since been heavily criticized [4]. The inclusion of two fat depots as one single entity may not reflect the separate functions and clinical implications of each adipose tissue. Indeed, recent studies focusing on separating EAT and PAT concluded that EAT alone was involved in the corresponding disease [5,6]. Indeed, EAT is a metabolically active adipose tissue [1] compared to PAT. Its accumulation and subsequent inflammation add to cardiovascular risks, potentially impacting left ventricle (LV) diastolic dysfunction [7,8]. Even more recently, EAT overload has raised concern as a risk factor in generalized inflammation from COVID-19 [9,10]. It is now recognized that the amount of EAT is prospectively and independently associated with the number of coronary events in at-risk populations [11]. Consequently, reproducibly quantifying EAT is a major public health objective aiming at a better identification of patients at high CV risk. EAT can indeed be visualized from standard cardiac magnetic resonance imaging (MRI) images, but its analysis is currently not performed in clinical routine, because the necessary manual image segmentation is extremely time-consuming, and its measurement is not sufficiently standardized.

Different imaging modalities have been proposed to quantify EAT burden. Transthoracic echocardiography, a forefront modality in cardiology, was used to measured EAT thickness on the free wall of the right ventricle [12]. However, only a single distance measurement was used to estimate EAT volume, strongly limiting the precision of this method because EAT is irregularly distributed around the heart. Cardiac computed tomography (CCT) imaging has become the gold standard for the quantification of EAT volume [13]. In more recent studies, semi-automatic and deep learning methods have been implemented to achieve the EAT segmentation [14,15]. However, the requirement of high spatial resolution CCT led to the use of elevated ionizing radiation doses, which could be a risk for patients’ follow-up.

Cardiac MRI is a versatile tool than can measure cardiac function, morphology, perfusion and characterizes myocardial tissue in a single exam [16]. Cardiac MRI is also highly sensitive to fat, which has long been considered as an obstacle for myocardial visualization. Cardiac fat remains under-appreciated as a diagnostic feature of cardiac MRI. To specifically probe fat around the beating heart with MRI, one can use a dedicated acquisition technique such as water-suppressed MRI [17] or Dixon MRI (3D) [18,19]. Alternatively, EAT volume may also be measured from a routine stack of short-axis cine images [20]. EAT quantification is usually performed manually, which is a time-consuming and tedious task subjected to inter-observer variability. To help observers, first the cine temporal information could ease distinguishing EAT from paracardial fat. Indeed, EAT is attached to the myocardium and moves at pace with cardiac contraction and torsion, whereas PAT is only moderately pulled by the cardiac contraction and expansion. Second, while the pericardial fascia is not clearly visible on short axis views, which was often reduced to a thin line that may be blurred by partial volume effects, on four-chamber (4Ch) views, the pericardium is generally less affected by partial volume effects resulting in better visualization. As such, the four-chamber view is recommended for evaluating pericarditis [21] and is a frequent choice of orientation to quantify EAT, PAT, and pericardial fat [22,23,24,25,26,27]. Consequently, the EAT analysis in this study were based on quantification of its 2D area representation in 4Ch long-axis cine MRI views. To address specifically this kind of segmentation challenges, deep learning approaches have recently bloomed. Indeed, fully automated methods applied on routine images, such as cine MRI, could be rapidly translated to the clinics. Bard et al. [23] developed a deep learning method to quantify pericardial fat in 4Ch long-axis cine MRI and evaluated it on the UK BioBank dataset. However, the segmentation of pericardial fat (EAT + PAT) limits the evaluation of the distinct roles and clinical implications of epicardial fat compared to paracardial fat.

Thus, we propose here to segment the thin EAT area on 4Ch cine MRI multi-frame images using state-of-the-art fully convolutional networks (FCNs) for cardiac image segmentation, that were adapted to segment EAT, PAT, and cardiac ventricles. A specific database of 4Ch cine MRI spanning diabetic, obese, and healthy subjects was leveraged to train, validate, and evaluate proposed FCN networks.

## 2. Materials and Methods

### 2.1. Study Population

**Figure 1:**
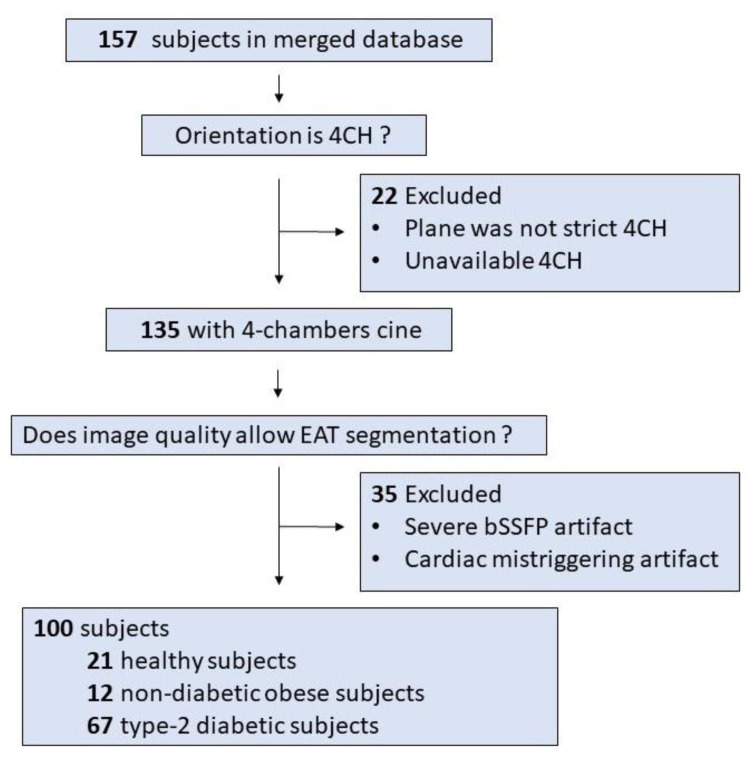
Overview of the study design.

A retrospective mono-centric database was defined totaling 153 subjects, out of which 100 exams could be exploited. The 100 enrolled subjects including healthy controls, type-2 diabetic patients, and non-diabetic obese patients were selected based on 4Ch orientation and the absence of severe artifacts as shown in Figure 1.

Patients were defined as having type 2 diabetes mellitus if they fulfilled any of the WHO criteria: HbA1c ≥ 6.5%, FBG level ≥ 7.0 mmol/L, oral glucose tolerance test result ≥ 11.1 mmol/L, or current treatment with antidiabetic agents. Obese non-diabetic patients were defined as the absence of any WHO criteria and a BMI ≥ 30 kg/m^2^. All enrolled subjects had normal left ventricular function, no history of heart failure or coronary heart disease.

### 2.2. MRI Acquisition

All subjects underwent cardiac MRI including the acquisition of a full stack of short-axis slices and a single slice four-chamber cine on a 3-T MRI system (Magnetom Verio, Siemens Healthineers, Erlangen, Germany) with a dedicated cardiac 32-channel coil array (Invivo, Gainesville, FL, USA). The cine series were acquired with a retrospectively ECG-gated balanced steady-state free precession (bSSFP) sequence with in-plane image resolution varying from 1.3 × 1.3 mm^2^ to 1.8 × 1.8 mm^2^ (depending on subjects), slice thickness of 6 mm, TE/TR = 1.2/3.2 ms, GRAPPA 2 (24 auto-calibration signal lines), temporal resolution of 28–35 ms, with 25 frames reconstructed. Further details of the cardiac MRI protocols were previously described [20,28,29,30,31]. N4 bias field correction [32] was applied to all image series before further processing.

### 2.3. EAT Segmentation

For reference, EAT volume was segmented by expert readers provided with full stack short-axis series using Argus viewer (Siemens Medical Solutions, Erlangen, Germany). In an independent session, two expert readers were provided with full 4Ch series and performed blinded segmentation of three labels using the FSLeyes viewer [33] (version 0.31, Paul McCarthy, University of Oxford, UK): heart ventricles (HV) (including both ventricle muscles and blood pools), epicardial (EAT), and paracardial (PAT) adipose tissues. EAT was defined as hyperintense signal within the pericardium around the ventricles. Peri-atrial fat was not included as it has been shown that peri-ventricle EAT alone had a stronger correlation with coronary diseases than total EAT [26]. All isles of periventricular fat were included to form EAT area. PAT was defined as fat adjacent but outside the pericardium. Segmentations were performed on three cardiac phases determined by readers having the entire series at their disposal: first phase, peak systole, and late diastole. The three segmented masks were propagated to the remaining frames using an automatic label propagation algorithm based on non-linear registrations, as previously described [34] resulting in 25 images segmented per subjects. Series in the test dataset were segmented by both readers, and reader 1 repeated blinded segmentations 6 weeks later.

### 2.4. Network Architecture

Two different fully convolutional networks (FCNs) were investigated: U-Net [35] with 48 filters for the first layer and FCN developed by Bai et al. [36] with 48 filters for the first layer, later referenced as FCNB. These networks are based on an encoder–decoder structure but differ in their decoder structure. The encoder part processes an image of arbitrary size as input and applies convolutional layers for extracting image features while the decoder upsamples and combines low-resolution featured map to the original input resolution. The absence of a dense layer allows these networks to process images of various sizes.

**Figure 2:**
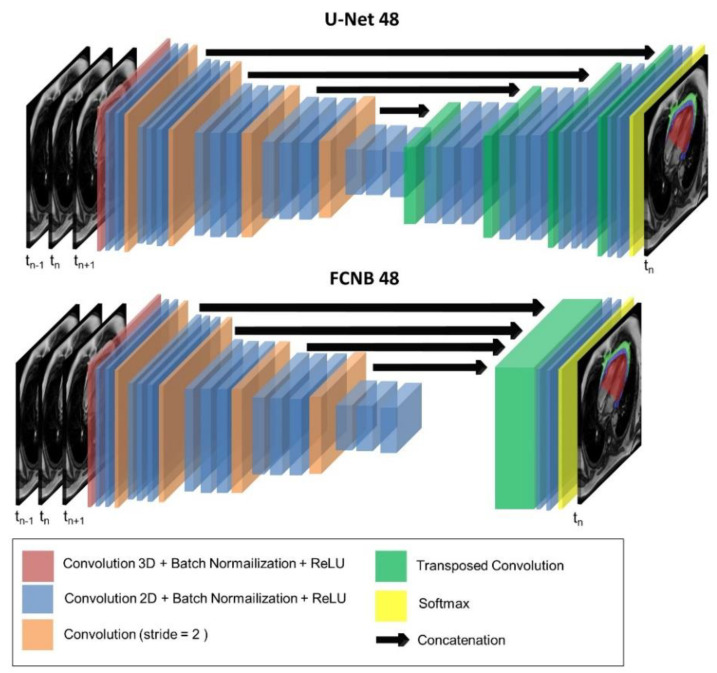
Networks’ optimized architecture. The two networks evaluated in this study: U-Net and fully-convolutional network (FCNB) architectures included a first 3D convolution layer to allow multiple cardiac frames as input. Following 2D convolution layers encoded images from 48 features up to 768 features. Eventually, the decoder targeted three labels for segmentation in the central input frame: epicardial adipose tissue (EAT), paracardial adipose tissue (PAT), and heart ventricles (HV).

The U-Net [35] has been the most popular 2D segmentation network for biomedical images and a fundamental component of many state-of-the-art cardiac image segmentation approaches [37,38,39]. The specificity of the U-Net is to employ skip connections between encoder and decoder to recover spatial information lost in downsampling layers as shown in Figure 2.

The second network investigated is the FCN developed by Bai et al. [36], later referred to as FCNB. FNCB has demonstrated excellent segmentation performances on the largest available cardiac MR dataset (UK-Biobank [40] Its specificity is based on the decoder that only consists of the concatenation of all featured maps, upsampled to the original resolution, as shown in Figure 2.

In their original papers, the cross-entropy loss was used to train those networks. However, this loss has shown limits to address class imbalance. In our study, regions of interest (ROI) were sparsely represented compared to the background and cross-entropy loss is inadequate to handle it. Thus, the loss function was defined as the mean dice between the probabilistic label map without background and the manually annotated label map.

### 2.5. Training

Specifically, optimized FCNB and U-Net were trained on three consecutive cine frames for segmentation of the central frame, providing a crucial temporal information often necessary for the experts to segment EAT. Input images were normalized to the range of [0,1] with fixed size (256 × 192 × 3), mask zero-padding or cropping was applied when needed.

For each batch (N = 30), on-the-fly data augmentation was performed using rotational transformation and/or image scaling before feeding them to the network. Both data augmentation were set using a random clipped normal distribution spanning from −30°/0.4 up to 30°/1.6 for rotational transformation and image scaling respectively. The Adam optimization [41] was used for minimizing the dice loss function with a constant learning rate of 1e-3. It took approximatively 35 min to train either the U-Net or FCNB on a Graphics Processing Unit (GPU) (NVidia Tesla K80).

The networks investigated were implemented using Python within the TensorFlow 2 framework. The FCNB model was adapted from the original implementation [42], whereas U-Net was custom-designed. To adapt to the proposed multi-frame approach, both 2D networks were modified to accept 2D+t inputs, considering the cardiac time dimension as a third dimension with limited horizon. Thus, the first convolution layer of each network was replaced with a 3D convolution layer with valid padding. The following layers were kept identical, processing extracted features independently of the input dimensions.

To perform a robust evaluation, networks were trained using cross-validation and evaluated on an independent dataset: the database was split in five subsets (500 images/20 subjects each reflecting our database populations distribution: 4 healthy controls, 13 type 2-diabetics, 3 nondiabetic obese patients). One subset (500 images) was used as a test set whereas the 4 other subsets were used for stratified cross-validation training, resulting in a 4-fold cross-validation. Thus, a single subset is retained as validation (500 images) whereas the 3 others (1500 images) are used for training, ensuring that validation and training dataset reflects the database population distribution.

### 2.6. Evaluation Metrics

Segmentation performances were evaluated for accuracy, propinquity, and surface estimation error. Dice similarity coefficient (DSC) measured segmentation accuracy from the overlap between the manual and automatic segmented surfaces (S_M_ and S_A_), defined as

(1) $$\mathrm{DSC}=2\;\frac{S_{M}\cap^{\;}S_{A}}{S_{M}+S_{A}}$$

The mean surface distance (MSD) calculated the propinquity between segmentations as is the mean distance (in mm) between segmented contours, defined as

(2) $$\mathrm{MSD}=\frac{1}{n_{M}+n_{A}}\left(\left(\overset{n_{M}}{\underset{k=1}{\sum }}d\left(k,S_{A}\right)\right)+\left(\overset{n_{A}}{\underset{k=1}{\sum }}d\left(k,S_{M}\right)\right)\right)$$

To evaluate the clinical final purpose, which is the quantitative measurement of EAT area, absolute relative surface error (RSE) was utilized, defined as

(3) $$\mathrm{RSE}=\frac{\left|S_{M}-S_{A}\right|}{S_{M}}$$

To further assess accuracy, positive predicted value (PPV) which is an indicator of over-segmentation (PPV << 1) was calculated on the entire database, defined as

(4) $$\mathrm{PPV}=\frac{S_{M}\cap^{\;}S_{A}\;}{S_{A}\;}$$

### 2.7. Statistical Analysis

Statistical analysis was conducted using R (version 3.6.3) [43]. Analysis of linear regression was used to study the correlation between manually evaluated EAT volume and 4Ch area. The metrics’ distribution normality was assessed using the Shapiro–Wilk test. Wilcoxon signed rank and Wilcoxon rank sum tests were used to investigate significant differences for each metrics between intra-inter observers and FCNs. To account for segmentation difficulty and clinical relevance [44] that scale with the quantity of EAT, networks’ performances were assessed per quartile of manually segmented EAT area (Q_1_ < 8.22 cm^2^ ≤ Q_2_ < 12.70 cm^2^ ≤ Q_3_ < 15.55 cm^2^ ≤ Q_4_).

## 3. Results

The selected 100 subjects were divided into three groups (21 healthy controls, 67 type-2 diabetic patients and 12 non-diabetic obese patients) as detailed in Table 1.

In studied database, EAT volumes spanned a wide range from 29 to 376 cm^3^, defining quartiles by: Q_1_ < 77.8 cm^3^ ≤ Q_2_ < 94.6 cm^3^ ≤ Q_3_ < 114.3 cm^3^ ≤ Q_4_.

**Figure 3:**
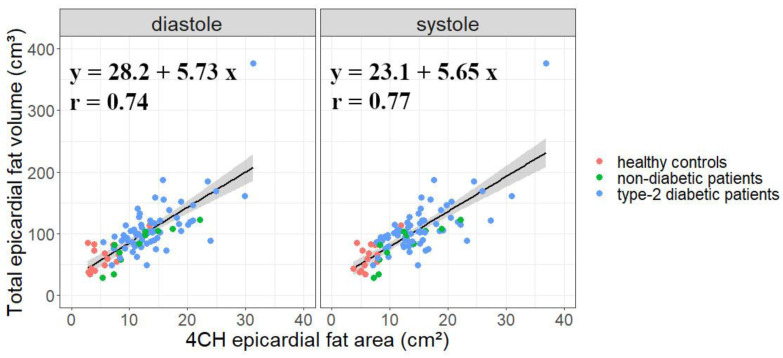
Comparison of reference total epicardial fat volume andproposed EAT area measured on four-chamber cine. EAT area was measured in end-systolic or end-diastolic frame across the 100 subjects’ database. The three cohorts merged for the database were identified by markers color.

Corresponding EAT areas as measured on 4Ch views correlated well with total EAT volume measured from the stack of short-axis cine (Figure 3) with a slightly higher correlation in systole (Pearson r = 0.77) than in diastole (Pearson r = 0.74). Thus, a wide range of EAT 4Ch areas was available from 1.2 cm^2^ to 37.2 cm^2^, with a lower range for healthy subjects from 2.5 to 13.7 cm^2^, from 1.2 cm^2^ to 23.2 cm^2^ for non-diabetic obese subjects and from 5.3 cm^2^ to 37.2 cm^2^ for type 2 diabetic patients.

As shown in Table 2, intra and inter-observer DSC confirmed excellent reproducibility for HV segmentation (DSC_Intra_ = 0.98 and DSC_Inter_ = 0.96 resp.). EAT and PAT differed between the two observers (DSC_Inter_ = 0.76 and 0.78 for EAT and PAT resp.), although segmentations performed twice by the same observer proved to be more reproducible (DSC_Intra_ = 0.83 and 0.85 for EAT and PAT resp.). Intra-observer DSC and MSD were significatively lower (*p* < 0.05) concerning EAT segmentation in the diastolic frame compared to the segmentation in the systolic frame. For inter-observer bias, differences in DSC, MSD, or RSE metrics were not statistically significant between diastolic and systolic frames.

FCNB and U-Net segmentations performance measured by DSC, were significantly lower (*p* < 0.05) than intra-observer bias for all labels (for EAT: DSC_Intra_ = 0.83, DSC_U-Net_ = 0.77, DSC_FCNB_ = 0.76). Both networks provided equivalent DSC, MSD, and RSE performance than inter-observer bias for all labels (for instance PAT: DSC_Inter_ = 0.78, DSC_U-Net_ = 0.80, DSC_FCNB_ = 0.78).

**Figure 4:**
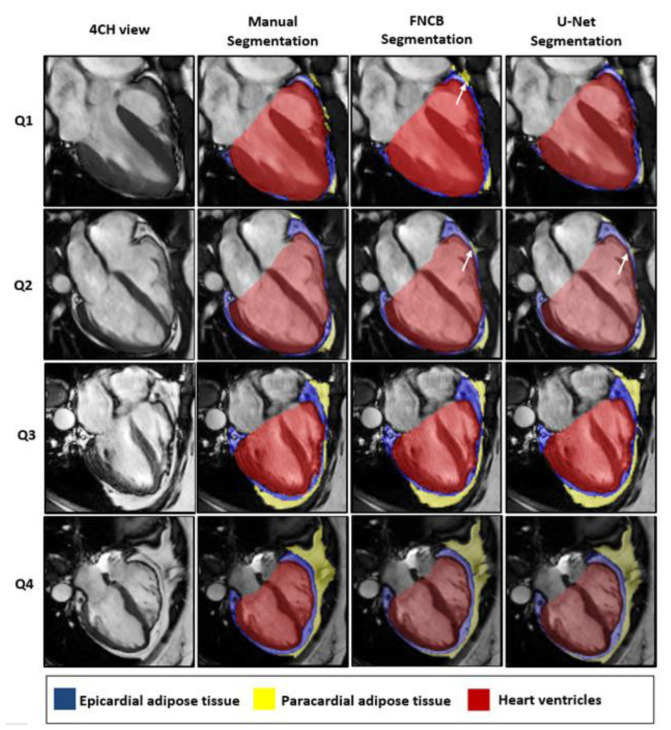
Representative segmentation results for each population defined by quartile of EAT area. Images were cropped around the heart for visualization. White arrows point out discrepancies between manual and automatic segmentations. As detailed in the methods, only periventricular EAT was segmented.

Across the four quartiles of data defined by equally populated ranges of EAT areas, both networks provided reliable segmentation of the heart ventricles (HV, FCNB: DSC_Q1-Q4_ = 0.97–0.96, U-Net: DSC_Q1-Q4_ = 0.97) as shown in Table 3. Interestingly, the network performances to segment EAT strongly depended on the population quartile. Indeed, U-Net DSC was significantly higher (*p* < 0.001) for upper quartiles as observed using U-Net: DSC_Q4_ = 0.83 > DSC_Q3_ = 0.80 > DSC_Q2_ = 0.76 > DSC_Q1_ = 0.69 as illustrated in Figure 4. DSC and RSE metrics demonstrated a gap of segmentation quality between the lower two quartiles and the upper two quartiles for both PAT and EAT segmentation (for EAT FCN: RSE_Q4_ = 15.60, RSE_Q3_ = 15.87 < RSE_Q2_ = 21.91 < RSE_Q1_ = 27.98). Across all quartiles, both networks had more difficulty separating PAT from EAT than identifying total pericardial fat (EAT+ PAT) in the image (with U-Net, RSE_EAT + PAT_ << RSE_EAT_ or RSE_PAT_ for all quartiles).

Over the database and for all labels, U-net outperformed (*p* < 0.0001) FCNB for segmenting accurately (DSC), nearer to the ground truth (MSD), thus providing a more reliable (i.e., accurate) measurement (RSE).

**Figure A1:**
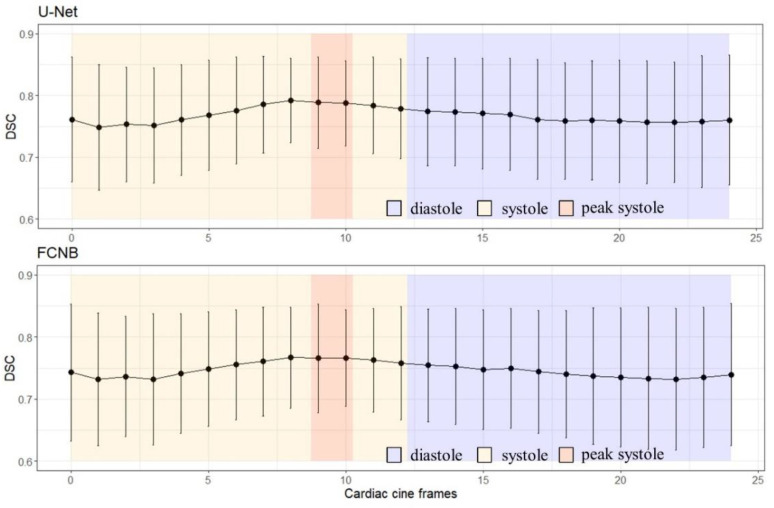
FCNs DSC performance varies along the cardiac cine frames with a maximum mean Dice and minimum standard deviation in systole.

FCNB and U-net performed significantly better (*p* < 0.05) for segmenting EAT area on the systolic frame compared to the diastolic frame (DSC_UNet-diastole_ = 0.76 DSC_UNet-systole_ = 0.80). These differences were not significant in PAT (see Appendix A Figure A1).

**Figure 5:**
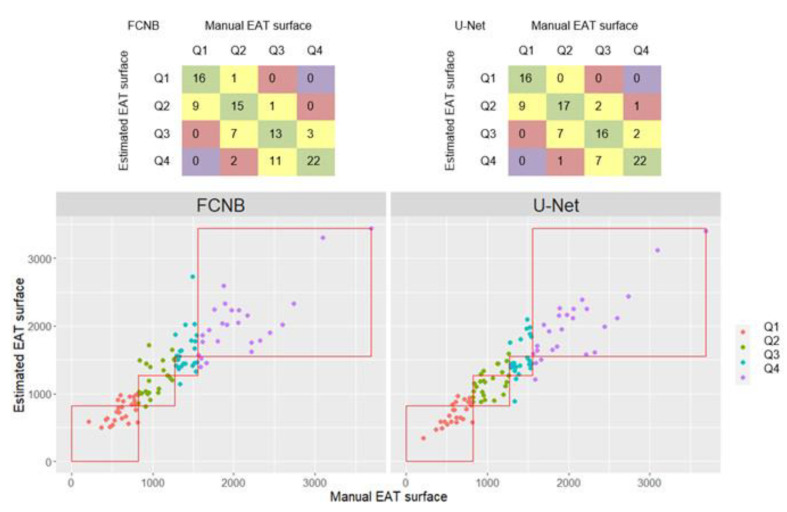
Quartile classification results from EAT area estimated from networks segmentation against classification from manual EAT area. Only segmentations from preferred systolic frames were shown here. Markers colors were defined by manual EAT quartiles. Red squares delineate manual EAT quartiles.

Classification of our database split by quartile of EAT burden was observed by confusion matrices. From Figure 5, the confusion matrices diagonal (in green) gave a measure of correct classification (66% for FCNB and 71% for U-Net), whereas the subdiagonal and the superdiagonal (in yellow) allowed evaluating a misclassification by one quartile (32% for FCNB and 27% U-Net) and the second subdiagonal and superdiagonal (in red) gave an estimate of a misclassification by two quartiles (2% for FCNB 2% for U-Net). As shown by subdiagonal confusion matrices and confirmed by PPV, FCNB significantly over-estimated EAT area compared to U-Net (PPV_FCNB_ = 0.73 < PPV_U-Net_ = 0.75, *p* < 0.0001).

## 4. Discussion

This study aimed at providing a rapid and fully integrable evaluation of epicardial fat burden. To achieve this evaluation, automated segmentation of the EAT layer was performed on four-chamber cine MRI series using Deep Learning approaches.

### 4.1. Four-Chamber-View Intrapericardial Fat Area Is a Relevant Measure of EAT

Confirming previous literature [24,25], the correlation found in this work between EAT area and volume across a wide range of EAT volumes (from 29 to 376 cm^3^) comforted the relevant use of four-chamber EAT area as a rapid but realistic measure of EAT burden. Already in past studies, the 2D EAT area has been linked to left ventricular diastolic dysfunction [22,26], hypertension and severity of insulin resistance [25], and non-alcoholic fatty liver disease patients [27]. Thus, four-chamber view holds potential as a surrogate to quantify EAT in routine clinical practice. Moreover, in four-chamber view, the pericardium beyond the apex of the heart could be visualized with more reliability. However, our database gathered retrospective studies in which EAT volume segmentation had been measured in short-axis views by different investigators over the years, which could lead to unaccounted volume imprecision. Ideally, the gold standard CCT EAT volume quantification would have been preferred but this examination is not commonly indicated for metabolic patients.

### 4.2. A Specific Database with Possible Extensions

This work leverages a unique database that combines a population spanning a large range of EAT quantity and manual segmentation of EAT on cine series. The strength of our dedicated database stands in its diversity in BMI, sex, age, health condition across many subjects (*n* = 100) (Table 1). Despite a large diversity of subjects, a disparity of age remains between younger healthy subjects and diabetic and/or obese patients. The addition of data from older healthy subjects, as well as elderly subjects (>65 years) would benefit the current database to reinforce our network training as elderly have been shown to be significantly more EAT burdened than younger individuals [45]. Our database could also be extended by including image sets from different MRI scanner types. Currently, this is a monocentric study and database. As a result, the trained models might not adapt well on datasets from scanners of different vendors and field strengths. Nevertheless, the database was made up of multiple protocols acquired over a decade, which already featured a variety of acquisition parameters and image quality levels. To further leverage the number of annotated data (2500 ground-truth, 25 images segmented per subject), generative adversarial network could be explored to extend beyond proposed data augmentation [46]. Another challenge are recurrent artifacts (aliasing, dark bands, flux artifacts) commonly observed in 3T bSSFP cine-MRI images, particularly pronounced in obese patients. This might preclude EAT segmentation and disturb networks accuracy. Training networks on artifacted images is another important addition to strengthen models for them to be ready for the clinic.

### 4.3. Challenge of EAT Segmentation

Experts and networks provided excellent results on large structures such as heart ventricles (DSC ≥ 0.96) and pericardial fat (DSC ≥ 0.88). However, one major challenge for the segmentation of EAT on cine MRI is to distinguish between burdening EAT and its extra-pericardial neighbor PAT. The pericardial fascia that separates those two fat compartments is about 2 mm thick [47,48] which is of the same order of magnitude as the image resolution (1.3–1.8 mm). This explains why both networks were able to segment combined EAT + PAT pericardial fat with appreciable precision, but the identification of individual fat was less satisfying. Nevertheless, FCN networks provided segmentation results on par with experts’ precision. Additionally, since cardiac contraction pulls onto the pericardium, its visualization improves in peak-systole [22], making this frame more suitable for the measurement of EAT when compared to diastole (p_intra_(DSCdia/DSCsys) = 0.0282).

One novelty has been to input multiple cardiac frames from the cardiac cycle to networks using a 3D first convolutional layer. It could be interesting in future work to enhance temporal information which is essential to detect the pericardial fascia. A map of cardiac deformations could enhance input images to be supplied to the network. It would be also interesting to investigate other network architectures, such as recurrent neural network, that could memorize information from adjacent slices to improve inter-slices coherence [49], but these extensions fall outside the scope of this work.

### 4.4. Comparing FCNs Performances

Specific complementary metrics (DSC, MSD, and RSE) have been chosen to evaluate EAT area segmentation and quantification. Alternatively, the Hausdorff distance metric is a common choice to evaluate segmentation performance [50], measuring the maximal pixel distance error between segmentations. However, EAT region is sparsely distributed around the heart, thus the Hausdorff distance was not considered in this work since it might range rapidly high, even when comparing two segmentations with similar areas.

**Figure A2:**
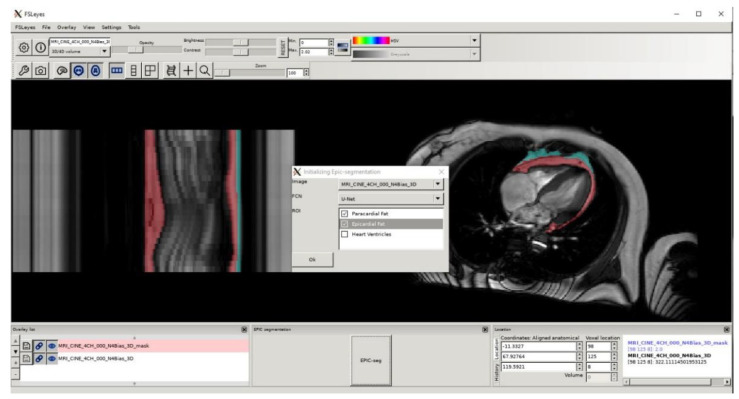
FSLeyes plugin interface for epicardial and paracardial segmentation will facilitate reproducibility of proposed methods.

From chosen metrics, U-Net outperformed FCNB for all labels, thus appearing preferrable to quantify EAT 4Ch area. Alternative semi- and fully automatic methods have been proposed for the EAT quantification on MRI-cine. Cristobal-Huerta et al. [51] developed an automatic pipeline composed of Law texture filters, snakes and K-cosine curvature analysis to partially quantify EAT volume, albeit on 10 subjects only. In a semi-automatic processing, Fulton et al. [52] applied landmarks on short-axis images from 12 subjects to unroll images into polar coordinates before employing a neural network for detection of epicardial fat contours. We were unable to compare our results with those previous works as segmentation metrics (e.g., DSC metric or Jaccard similarity index) were not provided. Recently, automatic total pericardial fat quantification has been developed in 4Ch cine MRI. Bard, Raisi-Estabragh et al. [23] obtained segmentation performances (DSC _EAT+PAT_ = 0.8) very similar to ours (DSC _EAT+PAT_ = 0.88) on their respective test-set. In their study, only the end-diastolic frame had been segmented while we segmented the full 4Ch cine MRI and trained on three consecutive cine frames to leverage cine temporal information. Finally, the optimized multi-frame U-Net was integrated in a FSLeyes plugin made available to the community [53] allowing comparison with further work and providing clinicians with a rapid EAT area segmentation (see Appendix A Figure A2).

### 4.5. Performances across Quartiles

Splitting the database in quartiles of EAT enabled to differentiate segmentation performances depending on EAT area. Indeed, segmentations quality from FCNs proved to be degraded in group Q1, in which EAT (as well as PAT) was thin and sparse as illustrated in Figure 4. However, EAT segmentations were on a par with inter-observers’ manual segmentation for the three upper quartiles and remained relevant for identifying patient at risk (Q_2_, Q_3_, Q_4_ ≥ 8.22 cm^2^) by measuring their EAT burden within 14% and 18% precision for U-Net and FCNB respectively. 

## 5. Conclusions

This study provides a methodology for fully automated segmentation of epicardial fat on multi-frame cardiac cine MRI, demonstrated across 100 subjects exhibiting low to high EAT quantities. EAT is often overseen in diagnosis but has received increasing attention as a relevant biomarker of cardiac risk. Automatic EAT evaluation could help to identify patients at risk, especially for diabetic patients. The comparison with EAT volume supports the potential of four-chamber cine EAT area as a surrogate for clinical evaluation, with higher segmentation robustness in systolic frame. Between the two FCNs investigated, the optimized U-Net was better suited to provide EAT area estimation with a 14.2% precision for the clinically relevant upper three quarters of targeted EAT range. EAT evaluation on cine, leveraging multi-frame information, could be further integrated to explore both retrospective and prospective cardiac studies without the need for a specific acquisition thanks to publicly provided automatic EAT area segmentation.

## Figures and Tables

**Table 1:** Study population clinical characteristics.

		Healthy	Non-Diabetic Obese	Type-2-Diabetic
**Clinical characteristics**				
	Number of participants	21	12	67
	Age, years	25 ± 10	41 ± 13	53 ± 10
	Gender: female, *n* (%)	11 (52)	10 (83)	41 (61)
	BMI, kg/m²	21.9 ± 2.6	40.8 ± 5.9	35.6 ± 6.8
**T2D**				
	Duration of diabetes, years			8 ± 6
**Cardiovascular risk factors, n (%)**				
	Hypertension	6 (29)	1 (8)	32 (48)
	Dyslipidemia	2 (10)	1 (8)	36 (54)
	Current Smoker, *n* (%)	3 (14)	1 (8)	8 (12)

**Table 2:** Mean values and standard deviations (in parenthesis) of segmentation results on the test set.

	DSC				MSD (mm)				RSE (%)			
	Intra	Inter	U-Net	FCNB	Intra	Inter	U-Net	FCNB	Intra	Inter	U-Net	FCNB
Paracardial Fat (PAT)	0.85 (0.06)	0.78 (0.09)	0.80 (0.08)	0.78 (0.10)	1.15 (0.63)	2.08 (1.49)	2.38 (1.78)	2.29 (1.47)	11.78 (8.09)	20.43 (18.77)	14.29 (10.44)	17.43 (17.50)
Epicardial Fat (EAT)	**0.83** **(0.07)**	**0.76** **(0.10)**	**0.77** **(0.07)**	**0.76** **(0.07)**	**1.53** **(1.32)**	**2.65** **(2.98)**	**1.71** **(1.06)**	**2.06** **(1.96)**	**13.02** **(14.59)**	**17.67** **(15.07)**	**20.33** **(15.70)**	**20.97** **(15.66)**
Pericardial Fat (EAT + PAT)	0.90 (0.04)	0.88 (0.05)	0.88 (0.06)	0.88 (0.06)	1.12 (0.66)	1.55 (0.07)	1.36 (0.90)	1.60 (1.28)	6.92 (7.16)	9.20 (6.80)	7.36 (9.40)	8.92 (12.97)
Heart ventricles (HV)	0.98 (0.01)	0.96 (0.02)	0.97 (0.02)	0.96 (0.03)	0.96 (0.5)	1.88 (2.24)	1.33 (0.79)	1.42 (0.89)	2.33 (2.20)	3.69 (3.18)	3.88 (4.46)	4.22 (5.80)

Metrics are reported as mean values (standard deviation). Systole and diastole segmentations were not differentiated in these metrics. DSC, dice similarity coefficient; MSD, mean surface distance; RSE, absolute relative surface error. Epicardial Fat values are highlighted in bold.

**Table 3:** DSC, MSD, RSE metrics evaluated per quartile (Q1-Q4) of EAT area for U-Net and FCNB.

**Q1**	**DSC**		**MSD (mm)**		**RSE (%)**	
	**U-Net**	**FCNB**	**U-Net**	**FCNB**	**U-Net**	**FCNB**
Paracardial Fat (PAT)	0.55	0.53	5.82	5.69	36.21	38.54
Epicardial Fat (EAT)	0.69	0.67	2.14	2.21	22.15	27.98
Pericardial Fat (EAT + PAT)	0.78	0.77	1.60	1.78	2.08	2.65
Heart ventricles (HV)	0.97	0.97	1.12	1.35	12.59	16.19
**Q2**	**DSC**		**MSD (mm)**		**RSE (%)**	
	**U-Net**	**FCNB**	**U-Net**	**FCNB**	**U-Net**	**FCNB**
Paracardial Fat (PAT)	0.76	0.75	2.68	2.82	17.29	20.83
Epicardial Fat (EAT)	0.76	0.74	1.22	1.53	17.85	21.91
Pericardial Fat (EAT + PAT)	0.87	0.87	1.16	1.35	7.55	8.60
Heart ventricles (HV)	0.97	0.97	1.11	1.65	2.57	3.04
**Q3**	**DSC**		**MSD (mm)**		**RSE (%)**	
	**U-Net**	**FCNB**	**U-Net**	**FCNB**	**U-Net**	**FCNB**
Paracardial Fat (PAT)	0.82	0.82	2.26	1.99	12.72	12.14
Epicardial Fat (EAT)	0.80	0.79	1.30	1.47	13.49	15.87
Pericardial Fat (EAT + PAT)	0.90	0.90	1.37	1.43	5.86	5.28
Heart ventricles (HV)	0.97	0.97	1.08	1.50	2.54	3.07
**Q4**	**DSC**		**MSD (mm)**		**RSE (%)**	
	**U-Net**	**FCNB**	**U-Net**	**FCNB**	**U-Net**	**FCNB**
Paracardial Fat (PAT)	0.80	0.78	2.46	3.12	13.65	16.72
Epicardial Fat (EAT)	0.83	0.79	1.40	2.06	11.72	15.60
Pericardial Fat (EAT + PAT)	0.91	0.90	1.40	1.84	5.64	6.43
Heart ventricles (HV)	0.97	0.96	1.31	2.60	3.20	4.52

Epicardial Fat values are highlighted in bold.

## Data Availability

The raw data supporting the conclusions of this article will be made available by the authors, without undue reservation.
